# Supplementary material for: Stemphylium lycopersici Nep1-like Protein (NLP) Is a Key Virulence Factor in Tomato Gray Leaf Spot Disease
Source: J Fungi (Basel). 2022 May 18;8(5):518. doi: 10.3390/jof8050518 (PMC9144795; doi:10.3390/jof8050518)
Supplement: Supplementary file 1 [file jof-08-00518-s001.zip › jof-1681581-supplementary.pdf]

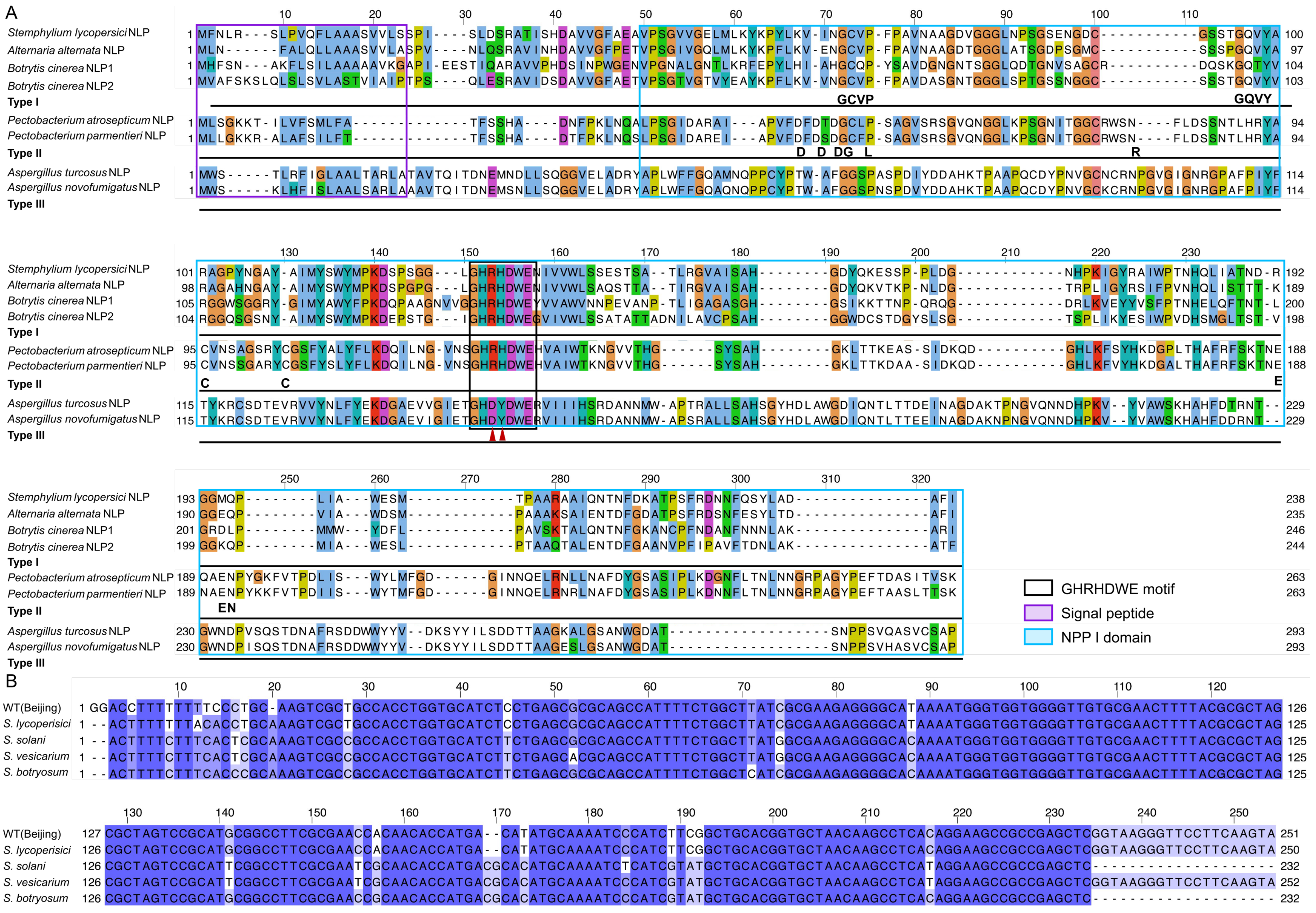
A
Stemphylium lycopersici NLP
Alternaria alternata NLP
Botrytis cinerea NLP1
Botrytis cinerea NLP2
Type I
GCVP
GQVY
Pectobacterium atrosepticum NLP
Pectobacterium parmentieri NLP
Type II
D D DG L
R
Aspergillus turcosus NLP
Aspergillus novofumigatus NLP
Type III
C C
E
EN
GHRHDWE motif
Signal peptide
NPP I domain
B
WT(Beijing)
S. lycoperisici
S. solani
S. vesicarium
S. botryosum

Figure S1 . Alignment of NLP proteins shows that *S. lycopersici* NLP is a type I NLP protein. (**A**) The *S. lycopersici* NLP protein sequence was aligned with typical type I, type II, and type III NLP proteins. Purple and blue boxes represent the signal peptide and NPP I domain, respectively. The black box highlights the most conserved heptapeptide motif (GHRHDWE) in the NPP1 domain; red triangles indicate the two amino acids (RH) which were conserved in type I and type II but divergent (DY) in type III NLPs. The conserved DxDxDG motif specific to type II NLPs is also noted. (**B**) A nucleotide alignment use *EF1α* gene in *Stemphylium* spp. to confirm the WT strain is a *S. lycopersicis* strain. The alignment was performed by the Jalview with Muscle method (http://www.jalview.org, accessed on 15 May 2022). *EF1α* gene sequence of WT (Beijing) strain used in this study, *S. lycopersici*, *Stemphylium solani*, *Stemphylium vesicarium,* and *Stemphylium botryosum* f. sp. *Lycopersici* were aligned.

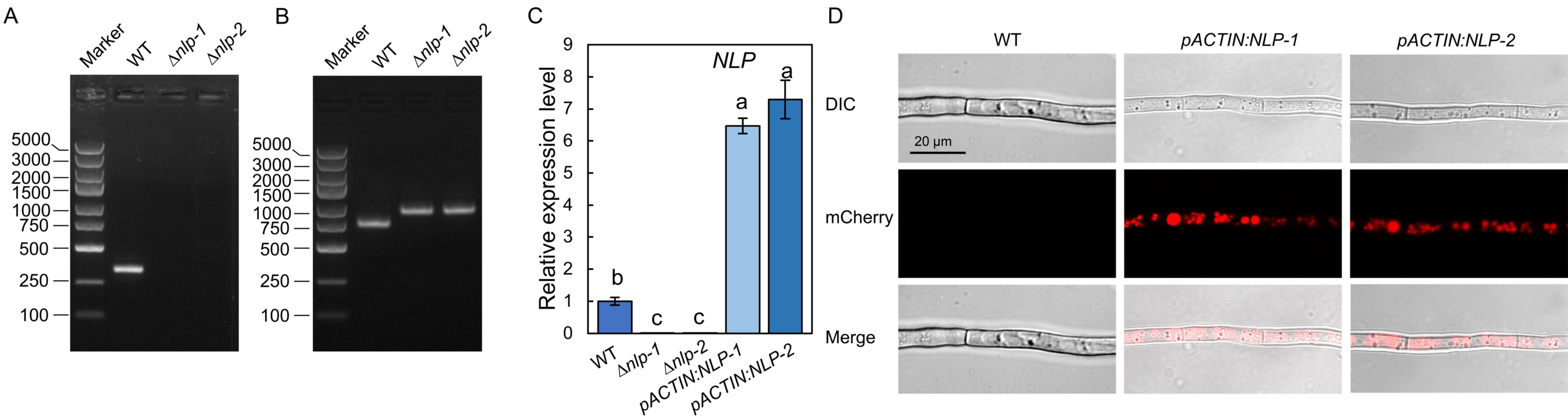

Figure S2. Confirmation of *NLP* gene knockout and overexpression *S. lycopersici* strains. (**A**) PCR results showing the targeted replacement of *NLP* gene in Δ*nlp-1* and Δ*nlp-2*. (**B**) PCR results of full-length amplification with primers (NLP-full-check) designed outside the gene indicate the length of *NLP* gene has changed in Δ*nlp-1* and Δ*nlp-2*. (**C**) Relative expression of *NLP* gene in the WT, Δ*nlp-1*, Δ*nlp-2*, *pACTIN:NLP-1*, and *pACTIN:NLP-2* strains determined by RT-qPCR. (**D**) mCherry fluorescence in *pACTIN:NLP* strains. Lowercase of a, b, and c denotes significant difference among multiple groups ($p < 0.05$) by Duncan's new multiple range test.

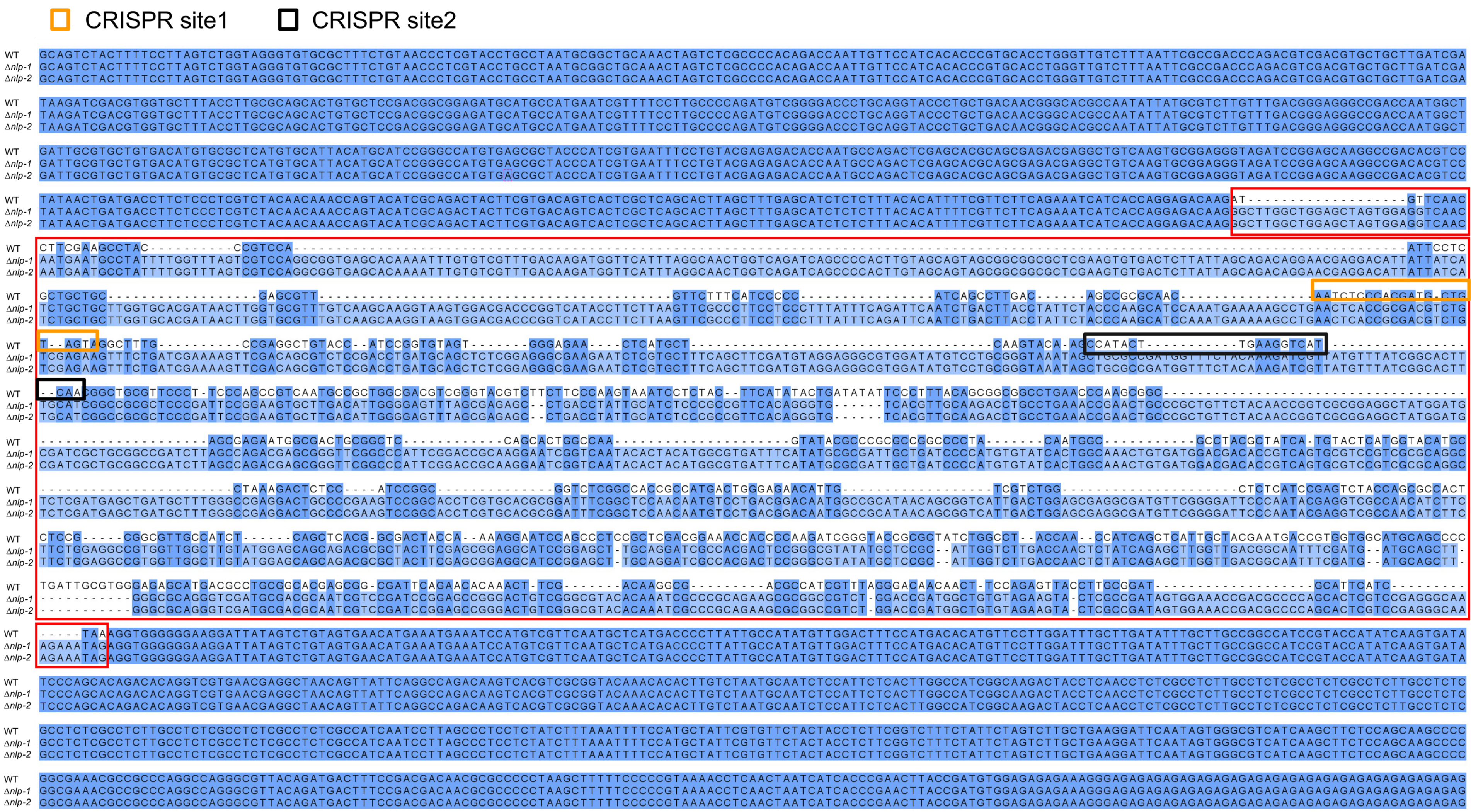

Figure S3. Confirmation of *NLP* gene knockout strains by sequence alignment. The red box represents the regions where the *NLP* gene has been replaced by the hygromycin B resistance marker gene (*hph*). The yellow and dark boxes represent CRISPR site 1 and 2 respectively.

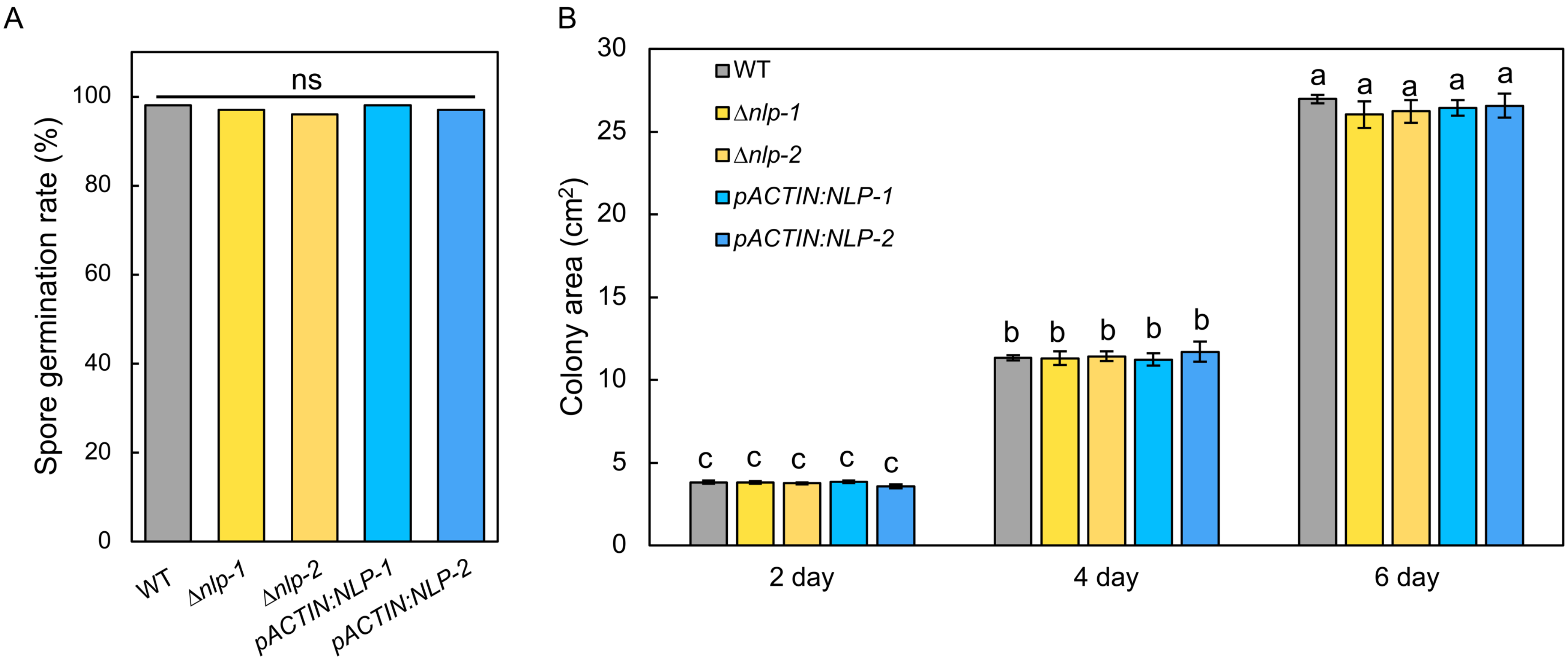

Figure S4. NLP does not affect spore germination and colony growth. (**A**) Conidial germination rate of the WT, *NLP* gene knockout mutants, and overexpression strains. (**B**) Colony growth of WT, *NLP* gene knockout mutants, and overexpression strains. Strains were grown on CM medium and colony growth was measured at days 2, 4, and 6 after inoculation. Statistical analyses were conducted using the DPS software; a, b, and c designate statistically significant differences. Lowercase of a, b, and c denotes significant difference among multiple groups ($p < 0.05$) by Duncan's new multiple range test. ns denotes no significant differences.

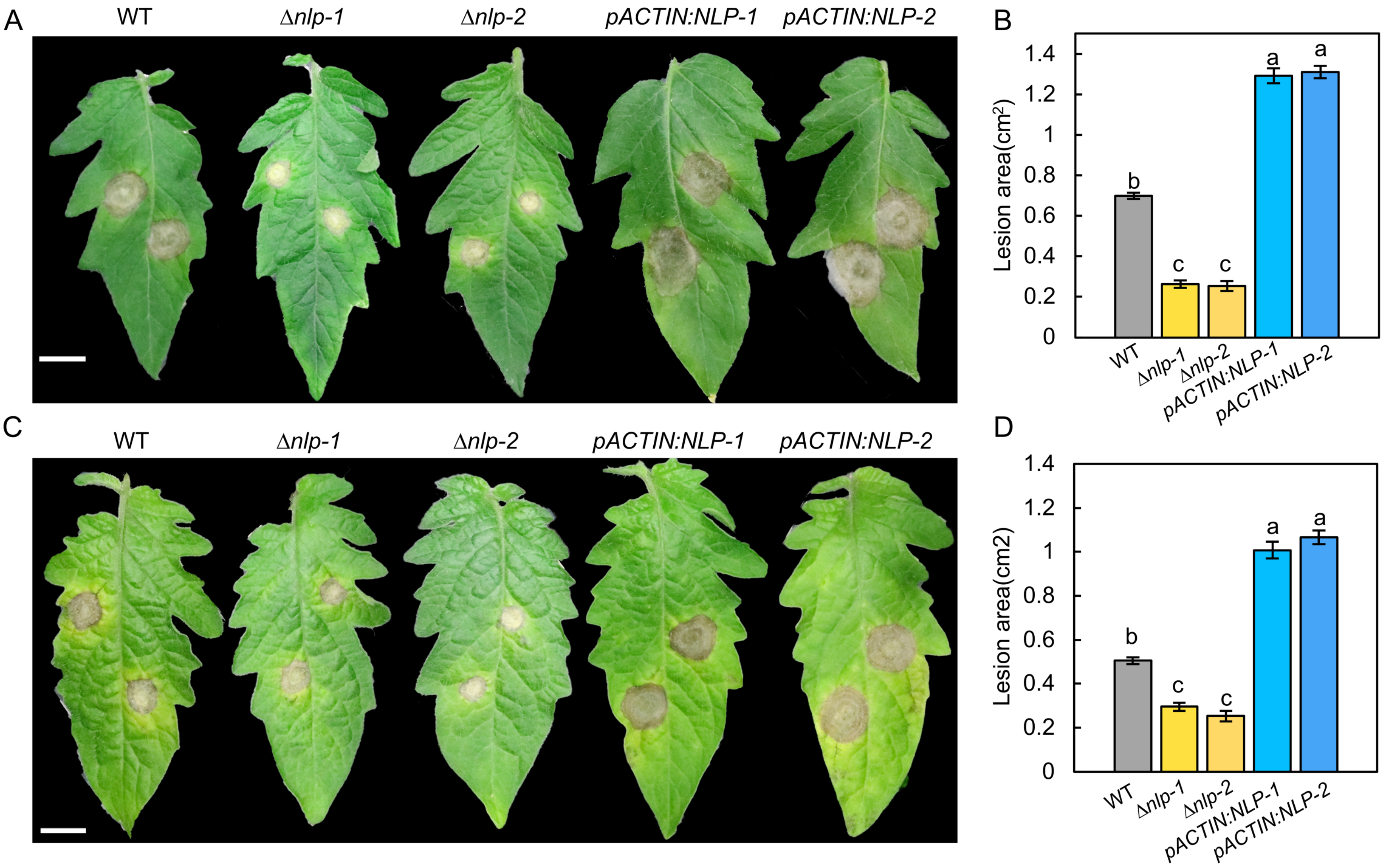

Figure S5. NLP is is a key virulence factor of *S. lycopersici* during infection on other tomato cultivars. (**A**) Infected leaves of tomato cultivar AC (Ailsa Craig) by the WT, Δ*nlp*, and overexpression strains at 5 days post inoculation (dpi). (**B**) Lesion area of tomato leaves of AC resulting from *S. lycopersici* infection of WT, Δ*nlp*, and overexpression strains. (**C**) Infected leaves of tomato cultivar E6203 by the WT, Δ*nlp*, and overexpression strains at 5 days post inoculation (dpi). (**D**) Lesion area of tomato leaves (E6203) resulting from infection of WT, Δ*nlp*, and overexpression strains. Lowercase of a, b, and c denotes significant difference among multiple groups ($p < 0.05$) by Duncan's new multiple range test.

Table S1 Primers for fungi NLP identification and gene manipulation

ID	Primers sequences
ITS1	TCCGTAGGTGAACCTGCGG
ITS4	TCCTCCGCTTATTGATATGC
EF1-α-F	CATCGAGAAGTTCGAGAAGG
EF1-α-R	TACTTGAAGGAACCCTTACC
gpd-F	CAACGGCTTCGGTCGCATTG
gpd-R	GCCAAGCAGTTGGTTGTGC
NLP U6-1 -crispr -F	TTTATTGAGCTTCTCTTCGTCTCCCACGATGCTGTAGTGTTTTAGAGCTAGAAATAGCA
NLP U6-1 -crispr -R	CTATTTCTAGCTCTAAAACTTGATGACCTTCAAGTATGCGAAGAGAAGCTCAATAAAGT
NLP-left-F	GGAGGCAGTTTGGATGAATGAC
NLP-lef-R	CTTGTCTCCTGGTGATGATTTCTG
NLP-right-F	AGGTGGGGGGAAGGATTATAGT
NLP- right-R	AAAGTCTGCGGGGAGAGGTG
NLP-TG-check-F	AGCGTTGTTCTTTCATCCCC
NLP-TG-check-R	AGCCGCAGTCGCCATTCT
NLP-full-check-F	ACTACTTCGTGACAGTCACTCGCTC
NLP-full-check-R	CGACATGGATTTCATTTCATGTTCACTACAG
Fungi-NLP-OE-F	TGAGAGAGCCCGTTTCTGACAGCTCTGCAG ATGTTCAACCTTCGAAGCCTAC
Fungi-NLP-OE-R	CATGTTATCCTCCTCGCCCTTGCTCACCATGATGAATGCATCCGCAAGGT

Table S2 Primers for *NLP* gene overexpression in tomato

ID	Primers sequences
Tomato-NLP-OE-F	ACAAGTTTGTACAAAAAAGCAGGCTTCCTCGAGATGTTCAACCTTCGAAGCCTACC
Tomato-NLP-OE-R	ACCACTTTGTACAAGAAAGCTGGGTTGAATTCGATGAATGCATCCGCAAGGTA

Table S3 Primers Used for RT-qPCR

ID	Primers sequences
NLP-qRT-F	GCTCACGGCGACTACCAAA
NLP-qRT-R	TGCTCTCCCACGCAATCAG
Fungi-ACTIN-qRT-F	TCCGTGACATCAAGGAGAAGC
Fungi-ACTIN-qRT-R	CAAGACAGAAGGCTGGAAAAGA
Tomato-ACTIN2-qRT-F	TTGCTGACCGTATGAGCAAG
Tomato-ACTIN2-qRT-R	GGACAATGGATGGACCAGAC
PR-STH2-qRT-F	GAAGGGGATCCATTGGGACAA
PR-STH2-qRT-R	TTCCCATAGCACTATCTTTTCCA
ERF.C3-qRT-F	TGCTGAAGGATCATCGCAAG
ERF.C3-qRT-R	ACCTAGCCATACACGAACACC
